# Antibiogram of Pseudomonas aeruginosa and Methicillin-resistant Staphylococcus aureus in patients with diabetes

**DOI:** 10.12669/pjms.304.4755

**Published:** 2014

**Authors:** Rubina Sabir, Syed Faraz Danish Alvi, Asher Fawwad, Abdul Basit

**Affiliations:** 1 Rubina Sabir, Laboratory Manager, Clinical and Research Laboratory, Baqai Institute of Diabetology and Endocrinology, Baqai Medical University, Plot No. 1-2, II-B, Nazimabad No2, Karachi-74600, Pakistan.; 2 Syed Faraz Danish Alvi, MBBS, Research Officer, Research Department, Baqai Institute of Diabetology and Endocrinology, Baqai Medical University, Plot No. 1-2, II-B, Nazimabad No2, Karachi-74600, Pakistan.; 3 Asher Fawwad, MBBS, M.Phil, Assistant Professor, Research Department, Baqai Institute of Diabetology and Endocrinology, Baqai Medical University, Plot No. 1-2, II-B, Nazimabad No2, Karachi-74600, Pakistan.; 4 Abdul Basit, FRCP, Professor of Medicine, Department of Medicine, Baqai Institute of Diabetology and Endocrinology, Baqai Medical University, Plot No. 1-2, II-B, Nazimabad No2, Karachi-74600, Pakistan.

**Keywords:** *Pseudomonas aeruginosa*, *Methicillin-resistant Staphylococcus aureus*, Antimicrobial drugs

## Abstract

***Objective: ***To determine the antibiogram of *Pseudomonas aeruginosa *and Methicillin-resistant *Staphylococcus aureus* (MRSA) in patients with diabetes.

***Methods:*** The study was carried out in the Microbiology Department of Clinical and Research Laboratory, Baqai Institute of Diabetology and Endocrinology (BIDE) from January 2012 to December 2012. All samples received in the laboratory were processed according to Clinical and Laboratory Standards Institute (CLSI) guidelines. Identification of Pseudomonas aeruginosa and Methicillin-resistant Staphylococcus aureus (MRSA) was done and antimicrobial susceptibility pattern was determined by disc diffusion method.

***Results:*** A total of 585 pathogens were isolated from 542 specimens of patients with diabetes. One hundred twenty one (20.68%) Pseudomonas aeruginosa and 25(4.27%) non-aeruginosa Pseudomonas were detected from 542 samples. Among 108 (18%) samples detecting the growth of Staphylococcus aureus, Methicillin-resistant Staphylococcus aureus (MRSA) were found in 42 (39%) samples. Pseudomonas aeruginosa showed marked susceptibility to imipenem (100%) followed by piperacillin / tazobactam (90.91%). All MRSA positive specimens were susceptible to vancomycin, but highly or completely resistant to the other antimicrobial drugs.

***Conclusion*** *: *In the present study imipenem, piperacillin/tazobactam and sulbactam-cefoperazone were found to be the most effective drugs against *Pseudomonas aeruginosa*. The majority of MRSA were resistant to one or more than one antimicrobial drugs. Vancomycin and imipenem were the most effective drugsagainst*Staphylococcus aureus* and MRSA. .

## INTRODUCTION

Diabetes is a metabolic disorder affecting 371 million people worldwide. At present, Pakistan has around 6.6 million people with diabetes; the number is anticipated to rise to 11.4 million by the year 2030.^1^ Patients with diabetes are more prone to life-threatening infections compared to patients without diabetes.^2^

*Pseudomonas aeruginosa,* a known opportunistic pathogen frequently causes serious infections. Usually compromised hosts like patients with diabetes are the main target of* Pseudomonas aeruginosa* and the pathogenicity of the organism is based on its ability to produce a variety of toxins, proteases and ability to resist phagocytosis. It may cause severe tissue damage in patients with diabetes and should never be ignored.^3^High frequency of *Pseudomonas aeruginosa* was found in different studies.^3^^-^^5^*Staphylococcus aureus* belongs to the family *Staphylococcaceae, *isspherical, Gram positive non motile cocci. *Staphylococcus aureus*is usually a component of mixed infections".^6^ Approximately 20–30% of the general population is "staph carriers".^7^ Methicillin-resistant *Staphylococcus aureus* (MRSA) are commonly seen among those who have weak immune system.^7^ MRSA may cause severe infections in hospitalized patients, such as bloodstream infections, surgical wound infections and pneumonia.^8^ The frequency of MRSA varied considerably in different studies i.e. from 31.1% in an Iranian study ^9^ to as high as 63.4% in a study conducted in China.^10^

The present study was designed to determine the antibiogram of *Pseudomonas aeruginosa* and Methicillin-resistant *Staphylococcus aureus* in patients with diabetes in a tertiary care hospital of Karachi-Pakistan.

## METHODS

A descriptive study was conducted at the Department of Microbiology, Clinical and Research Laboratory, Baqai Institute of Diabetology and Endocrinology (BIDE), a 24 hours laboratory service in Karachi-Pakistan from January 2012 to December 2012.

Bone, pus, body fluids and tissue samples received in the laboratory were inoculated on Blood agar (Oxoid), Chocolate agar (Oxoid), MacConkey agar (Oxoid) plates and Thioglycollate broth (Oxoid). Pus cultures were inoculated on Sabouraud Dextrose agar (Oxoid) plates. After inoculation all the plates were incubated overnight in previously set incubator at 35°C. Growth of organisms was observed and identification tests were done. *Pseudomonasaeruginosa *species were identified by Gram staining, motility, catalase, oxidase and pyocyanin production tests, while identification and confirmation of* Staphylococcus aureus* strains were done by colonial morphology on blood agar, Gram stain, haemolysis, catalase and coagulase tests.^11^^,^^12^

Antimicrobial susceptibility of *Pseudomonasaeruginosa *and *Staphylococcusaureus *was done by Kirby-Bauer disc diffusion method according to Clinical and Laboratory Standards Institute (CLSI) guidelines using various antibiotic discs (Oxoid).^3^^,^^5^^,^^9^ Isolated colonies of *Pseudomonasaeruginosa* and *Staphylococcusaureus* were inoculated on Mueller-Hinton agar (Oxoid) plates by achieving 0.5 McFarland turbidity standard.^9^ The antibiotic discs of clavulanic acid (AMC) 30 μg, cloxacillin (OB) 5 μg, piperacillin/tazobactam (TZP) 110 μg, cefotaxime (CTX) 30 μg, cefpirome (CPO) 30 μg, sulbactam-cefoperazone (SCF) 105 μg, vancomycin (VA) 30 μg, aztereonam (ATM) 30 μg, imipenem (IPM) 10 μg, gentamicin (CN) 10 μg, tobramycin (TOB) 10 μg, amikacin (AK) 30 μg, erythromycin (E) 15 μg, clarithromycin (CLR) 15 μg, clindamycin (DA) 2 μg, ciprofloxacin (CIP) 5 μg, chloramphenicol (C) 30 μg, sulphamethethoxazole (SXT) 25 μg, fosfomycin (FOS) 50 μg, fusidic acid (FD) 10 μg and doxycycline (DO) 30 μg, discs were used to assess the antimicrobial susceptibility. The inhibition zones were interpreted according to the CLSI guideline (CLSI, 2011). All *Staphylococcus* species were tested for Methicillin resistance using cloxacillin and oxacillin.^10^^,^^12^

***Statistical Analysis: ***Data analysis was done using Statistical Package for Social Sciences (SPSS), version 13.0. Data presented in the form of frequency and percentage.

## RESULTS

Overall, a total of 585 pathogens were isolated from 542 specimens of patients with diabetes. Tissue were the commonest specimens received in the laboratory 314 (57.93%) followed by pus 95 (17.5%), bone 32 (5.9%) and other samples (blood, urine, stool, sputum and body fluids) 101 (18.6%) as shown in Table-I.

The frequency of *Pseudomonas aeruginosa* was 27.07%, 24.21%, 37.50%, and 33.33% in tissue, pus, bone and fecal specimens respectively. About 5.41%, 5.26% and 9.38% tissue, pus and bone specimens respectively showed the growth of Non-aeruginosa *Pseudomonas* as shown in Table-I. Out of 131 (22%) Gram positive pathogens, *Staphylococcus aureus* was detected in 18%, *Streptococcus* species in 15%, coagulase negative *Staphylococci *in1.5% and* Streptococcus pyogenes *was found in 0.8% specimens.

Frequency of *Staphylococcus aureus* was 31.6%, 22.3%, 1.6% and18.8% in pus, tissues, bone and body fluids respectively. Methicillin resistant *Staphylococcus aureus* (MRSA) were found in 42 (39%) specimens including tissue 40%, pus 40% and bone 33.3% (Table-II).

Antimicrobial sensitivity patterns of microbial isolates are shown in Table-III. Both *Pseudomonas aeruginosa* and non-aeruginosa *Pseudomonas *showed 100% sensitivity against imipenem.

All Gram positive isolates were susceptible to vancomycin. *Staphylococcus aureus *showed highest susceptibility to imipenem (90%), piperacillin / tazobactam (70%), clavulanic acid (67%), cloxacillin (61%), amikacin and doxycycline (59%), sulbactam / cefoperazone (57%) and clindamycin (51%) while it demonstrated highly resistant pattern to chloramphenicol (88%), erythromycin (82%), fusidic acid (76%), fosfomycin and clarithromycin (70%), trimethoprim / sulphamethoxazole (69%), cefpirome (68%) and ciprofloxacin (60%) as shown in Table-III.

All MRSA pathogens showed susceptibility to vancomycin (100%), imipenem (81%) and doxycycline (55%) but were found highly resistant to cefpirome and erythromycin (98%), clarithromycin, chloramphenicol and ciprofloxacin (93%), fosfomycin, fusidic acid and trimethoprim / sulphamethoxazole (83%), sulbactam / cefoperazone (81%), clindamycin (76%), amikacin (67%) and piperacillin / tazobactam (60%) as shown in Table-III.

## DISCUSSION

The results of our study show a comprehensive evaluation of microbiological profile and antimicrobial susceptibility pattern of two super bugs *Pseudomonasaeruginosa *and Methicillin-resistant *Staphylococcus aureus* in patients with diabetes. 

**Table-I:** Frequency of *Pseudomonas aeruginosa, *Non-aeruginosa* Pseudomonas* and *Staphylococcus aureus*in different specimens (n= 542).

***Specimen*** ***n (%)***	***Pseudomonas aeruginosa *** ***n (%)***	***Non-aeruginosa Pseudomonas *** ***n (%)***	***Staphylococcus aureus *** ***n (%)***
*Tissue* 314 (57.93)	85 (27.07)	17 (5.41)	70 (22.3)
*Pus* 95 (17.53)	23 (24.21)	5 (5.26)	30 (31.6)
*Bone* 32 (5.90)	12 (37.50)	3 (9.38)	6 (18.8)
*Others* 101 (18.6)	---	---	2 (2)

Data presented as n (%)

**Table-II:** Frequency and Percentage of Methicillin-resistant* Staphylococcus aureus* (MRSA) in different clinical specimens

***Specimen***	***MRSA*** ***n***	***MRSA*** ***%***
Tissue (314)	28	40
Pus (95)	12	40
Bone (32)	2	33.3

Data presented as n (%)

**Table-III:** Antimicrobial sensitivity patterns of microbial isolates

***Antimicrobial agent***	***Pseudomonas aeruginosa***		***Non-aeruginosa Pseudomonas***		***Staphylococcus aureus***		***Methicillin-resistant Staphylococcus aureus***	
	***n (%)***		***n (%)***		***n (%)***		***n (%)***	
	***S***	***R***	***S***	***R***	***S***	***R***	***S***	***R***
AMC	38 (31.40%)	83 (68.60%)	9 (36%)	16 (64%)	72 (67%)	36 (33%)	0	42 (100%)
OB	----	----	----	----	66 (61%)	42 (39%)	0	42 (100%)
TZP	110 (90.91%)	11 (9.09%)	22 (88%)	3 (12%)	76 (70%)	32 (30)	17 (40%)	25 (60%)
CTX	29 (23.97%)	92 (76.03%)	6 (24%)	19 (76%)	----	----	----	----
CXM	10 (8.26%)	111 (91.7%)	5 (20%)	20 (80%)	----	----	----	----
CPO	58 (47.93%)	63 (52.07%)	18 (72%)	7 (28%)	35 (32%)	73 (68%)	1 (2%)	41 (98%)
SCF	107 (88.43%)	14 (11.57%)	23 (92%)	2 (09%)	62 (57%)	46 (43%)	8 (19%)	34 (81)
VA	----	----	----	----	108 (100%)	0	108 (100%)	0
ATM	95 (78.51%)	26 (21.49%)	16 (64%)	9 (36%)	----	----	----	----
IPM	121 (100%)	0	25 (100%)	0	97 (90%)	11 (10%)	34 (81%)	8 (19%)
CN	68 (56.2%)	53 (43.80%)	16 (64%)	9 (36%)	----	----	----	----
TOB	68 (56.2%)	53 (43.80%)	16 (64%)	9 (36%)	----	----	----	----
AK	98 (80.99%)	23 (19.01%)	21 (84%)	4 (16%)	64 (59%)	44 (41%)	14 (33%)	28 (67)
E	----	----	----	----	19 (18%)	89 (82%)	1 (2%)	41 (98%)
CLR	----	----	----	----	32 (30%)	76(70%)	3 (7%)	39 (93%)
DA	----	----	----	----	55 (51%)	53 (49%)	10 (24%)	32 (76%)
CIP	79 (65.29%)	42 (34.71%)	16 (64%)	9 (36%)	43 (40%)	65 (60%)	3 (7%)	39 (93)
C	----	----	----	----	13 (12%)	95 (88%)	3 (7%)	39 (93%)
SXT	19 (15.70%)	101 (84.30)	7 (28%)	18 (72%)	33 (31%)	75 (69%)	7 (17%)	35 (83%)
FOS	----	----	----	----	32 (30%)	76(70%)	7 (17%)	35 (83%)
FD	----	----	----	----	26 (24%)	82 (76%)	7 (17%)	35 (83%)
DO	----	----	----	----	64 (59%)	44 (41%)	23 (55%)	19 (45%)

S = sensitive, R = resistant, AMC = clavulanic Acid, OB = cloxacillin, TZP = piperacillin**/ ***tazobactam,*

CTX = cefotaxime, CXM = cefuroxime, CPO = cefpirome, SCF = sulbactam-cefoperazone,

VA = vancomycin, ATM = aztereonam, IPM = imipenem, CN = gentamicin, TOB = tobramycin,

AK = amikacin, E = erythromycin, CLR = clarithromycin, DA = clindamycin, CIP = ciprofloxacin,

C = chloramphenicol, SXT = sulphamethethoxazole, FOS = fosfomycin, FD = fusidic acid,

DO = doxycycline, Data presented as n (%).

High frequency of *Pseudomonas aeruginosa* was found in various studies.^4^^,^^5^^,^^13^In our study 20.68% specimens showed the growth of *Pseudomonas aeruginosa* similar to the findings of other studies.^14^^,^^15^ While low frequency was observed in studies conducted in Iran (5.4%)^9^ and Northern areas of Pakistan (4%).^16^

The frequency of Gram positive isolates in this study was 22%, while in a local study from Pakistan frequency of Gram positive was found to be 46%.^16^ May be the difference is due to environmental factors and the available public health facilities. 

Most frequent aerobic Gram positive isolate found in our study was *Staphylococcus aureus* (18%). The frequency of MRSA in the study was 39%, which is similar to the findings of other studies.^9^^,^^17^ However, studies conducted in China and India showed high prevalence of MRSA63.4% and 65.5% respectively.^10^^,^^18^

Imipenem was found to be the most effective drug against *Pseudomonas aeruginosa* in our study which is similar to the other studies.^9^^,^^13^^-^^15^^,^^19^^-^^21^ In this study piperacillin / tazobactam appeared as the second most effective drug against *Pseudomonasaeruginosa* infection (90.91% susceptibility). Whereas Indian studies showed 83% and 66% susceptibility of piperacillin / tazobactam towards *Pseudomonas aeruginosa*.^14^^,^^21^^,^^22^

Our study results showed 80.9% susceptibility of *Pseudomonasaeruginosa*isolates towards amikacin is also similar to the other studies.^14^^,^^15^^,^^22^The frequency of imipenem susceptibility to *Staphylococcus aureus* in diabetic foot infections was 90% in our study while it was 100% in an Indian study;^23^ whereas it was only 33.3% in an Iranian study.^15^

Highly resistant MRSA to cefpirome and erythromycin (98%) were observed in our study whereas it was 88.2% in a Chinese study,^10^ while lower rates (40%) were found in an Indian study.^24^ About 93% of MRSA showed resistance towards clarithromycin, chloramphenicol and ciprofloxacin in this study while in an Indian study 20% of chloramphenicol and 50% of ciprofloxacin resistant MRSA were isolated.^24^ In our study 83% of sulphamethoxazole resistant MRSA were found while they were 58.8% in Chinese study^10^ High rate of clindamycin resistant MRSA (82.4%) was found in Chinese study^10^ while it was 76% in our study.

## CONCLUSION

In the present study imipenem, piperacillin/tazobactam and sulbactam-cefoperazone were found to be the most effective drugs against *Pseudomonas aeruginosa*. The majority of MRSA were resistant to one or more than one antimicrobial drugs. Vancomycin and imipenem were the most effective drugs against *Staphylococcus aureus* and MRSA.

## Author Contributions:

*Rubina Sabir, Syed Faraz Danish Alvi, Asher Fawwad:* Substantial contributions to conception and design, or analysis and interpretation of data.

*Rubina Sabir, Syed Faraz Danish Alvi, Asher Fawwad,* *Abdul Basit:* Drafting the manuscript and revising it critically for important intellectual content and Final approval of the version to be published.

## Notes

***Declaration of Competing Interests: ***Nothing to declare.
